# Treatment of Severe Tricuspid Regurgitation with the TricValve System Implantation—Preliminary Results of a Prospective Registry

**DOI:** 10.3390/jcm14228103

**Published:** 2025-11-15

**Authors:** Adam Rdzanek, Maciej Dąbrowski, Ewa Pędzich, Mariusz Tomaniak, Piotr N. Rudziński, Agnieszka Kapłon-Cieślicka, Adam Piasecki, Janusz Kochman, Adam Witkowski, Piotr Scisło

**Affiliations:** 1 1st Department of Cardiology, Medical University of Warsaw, 02-091 Warsaw, Poland; 2 Department of Interventional Cardiology and Angiology, National Institute of Cardiology, 04-628 Warsaw, Poland; mdabrowski@ikard.pl (M.D.);; 3 Department of Coronary and Structural Heart Diseases, National Institute of Cardiology, 04-628 Warsaw, Poland

**Keywords:** caval valve implantation, tricuspid regurgitation, TricValve system

## Abstract

**Background**: Tricuspid regurgitation (TR) is a common valvular heart disease that often causes disabling symptoms. Caval valve implantation with the TricValve system is one of the transcatheter treatment options proposed for TR symptom reduction. With this prospective registry, we aim to summarize our early experience with TricValve system implantation. **Methods**: Registry participants, selected out of patients who were referred for TR treatment but who were not eligible for the transcatheter tricuspid edge-to-edge valve repair (T-TEER), were qualified for the caval valve implantation following a HeartTeam discussion. **Results**: Four patients (four women; median age 71 years; 67.5–77 years) in whom a one-year follow-up was completed were included in the study. The patients were highly symptomatic in the NYHA class III despite intensive diuretic treatment; all of them were considered a high-mortality risk during conventional cardiac surgery. The TricValve system was successfully implanted in all patients. At 6-month follow-up, we observed a reduction in symptoms in three out of four patients. Up to 12 months, only one patient survived, with a reduction in symptoms of NYHA class II; two patients died because of heart failure; one died due to a progression in neoplastic disease. **Conclusions**: In highly symptomatic TR patients who were not eligible for the T-TEER and who had a prohibitive risk of cardiac surgery, TricValve implantation led to a reduction in symptoms in a 6-month perspective. Long-term survival was limited mainly by heart failure progression and severe concomitant disorders. Further studies are needed to fully elucidate the role of caval valve implantation in the treatment of TR patients.

## 1. Introduction

Tricuspid regurgitation (TR) is one of the most common acquired valvular heart diseases.

Population studies indicate that the frequency of TR increases with age, and in elderly patients, it exceeds the prevalence of aortic stenosis [1]. In many patients, despite optimal medical treatment, TR can be highly symptomatic, reducing the patient’s quality of life, eventually leading to severe right ventricular failure. In recent years, several minimally invasive treatment options have been proposed in this population of patients. Tricuspid valve transcatheter edge-to-edge repair (T-TEER), based on the leaflet approximation for TR reduction, is the most commonly applied method, with confirmed effectiveness and a favorable safety profile [2]. Although commonly used, the T-TEER technique has several major limitations. Since the procedure is mainly guided by echocardiography, it requires very good image quality in the transesophageal examination; moreover, several anatomical features, such as a large coaptation gap, short and tethered leaflets, or the presence of accessory leaflets, may cause procedural failure. In highly symptomatic patients who are not optimal candidates for T-TEER, a different technique can be used. Caval valve implantation (CAVI) is based on heterotopic, angio-guided placement of two self-expanding valvular prostheses in the ostium of the superior cava (SVC) and inferior vena cava (IVC). Currently, a dedicated system—TricValve (P&F Products&Features GmbH), is approved for intervention. This prospective registry summarizes the preliminary experience with the first series of patients treated in our institutions with TricValve system implantation.

## 2. Materials and Methods

Out of the patients with severe TR, who were referred for transcatheter treatment to the Medical University of Warsaw and the National Institute of Cardiology, patients who were not optimal candidates for T-TEER were selected. A decision regarding T-TEER eligibility, based on common criteria [3] and local experience, was made by the echocardiographer and interventional cardiologist. In highly symptomatic patients who were disqualified from T-TEER, after a local heart team discussion, a CAVI procedure was considered. Computed tomography of the right heart and both SVC/IVC were performed and submitted for evaluation by the TricValve system manufacturer (P&F Products&Features GmbH, Vienna, Austria). Anatomically suitable candidates were offered the possibility of a CAVI procedure for right ventricular failure symptom reduction. The procedures and the data collection were accepted by the local ethics committees of both the Medical University of Warsaw and the National Institute of Cardiology. Due to the limited number of registry participants, the results are presented as a case series description.

## 3. Results

Between September 2021 and September 2024, overall, five patients were treated in the Medical University of Warsaw and the National Institute of Cardiology with TricValve system implantation. With this prospective registry, we present a summary of the first four patients (four women; median age 70.5; 67.5–74.0) in whom a one-year follow-up was completed. All patients were highly symptomatic in New York Heart Association (NYHA) class III despite intensive diuretic treatment, and all patients were considered a high-mortality risk during conventional cardiac surgery (median Euroscore II 9.7% (8.6–12.1%); median Triscore 35% (20–48%]). Patient characteristics and the CAVI procedure details are summarized in Table 1. At the 6-month follow-up, all patients remained alive, with three showing improvement in heart failure symptom severity. However, by 12 months, only one patient was still alive and subsequently classified as NYHA functional class II. Three patients had died: two due to worsening right heart and multiorgan failure, and one due to progression of neoplastic disease. A detailed description of the case series is presented below.

### 3.1. Case 1

**Figure 1:**
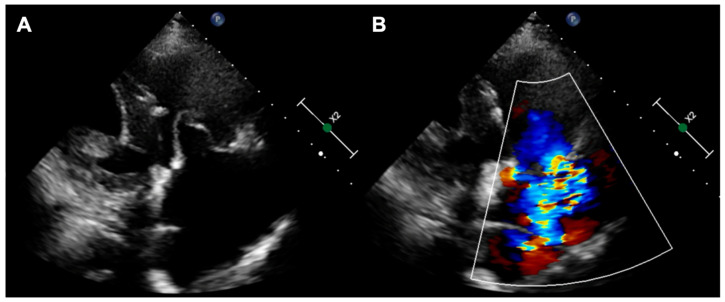
(**A**) Degenerated tricuspid CoreMatrix bioprosthesis; (**B**) torrential tricuspid regurgitation.

**Figure 2:**
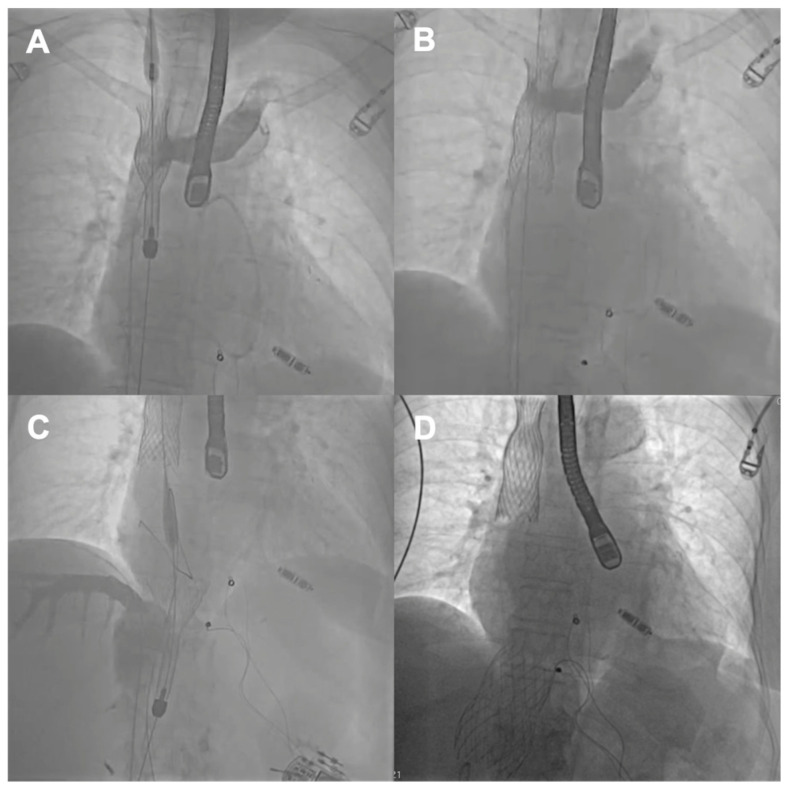
Consecutive steps of the TricValve procedure: (**A**) SVC stent positioning; (**B**) SVC stent implantation; (**C**) IVC stent positioning; (**D**) final angiographic result. SVC—superior vena cava; IVC—inferior vena cava.

The first patient was a 77-year-old woman who had persistent heart failure symptoms (HF) in NYHA class III due to torrential regurgitation of the tricuspid bioprosthesis (CoreMatrix) that was implanted five years before. (Figure 1) The patient’s past medical history included a myocardial infarction 15 years before, coronary artery bypass grafting 15 years before, permanent atrial fibrillation, peripheral artery disease, type 2 diabetes, chronic kidney disease, and implantation of a leadless pacemaker. The patient suffered from recurrent exacerbations of HF that required hospitalization despite intensive diuretic treatment in the preceding year. Due to the very high predicted mortality risk (Table 1) and the history of two previous cardiac surgeries, the patient refused surgical treatment. Because the anatomy of the degenerated tricuspid bioprosthesis was not suitable for edge-to-edge treatment, the patient was offered the CAVI procedure. Valves of the TricValve system were implanted in the superior cava (SVC) and inferior vena cava (IVC), respectively, 21 mm and 41 mm. (Figure 2) The procedure was complicated, with access site bleeding requiring surgical intervention. The patient’s echocardiography, post their procedure, revealed a mild paravalvular leak in the IVC valve. One month after the procedure, the patient persisted in NYHA functional class II; however, during the following months, she was hospitalized due to exacerbation of their right ventricular failure. Eventually, the patient died 9 months after the procedure because of the progressive worsening of right ventricular dysfunction and multiorgan failure.

### 3.2. Case 2

**Figure 3:**
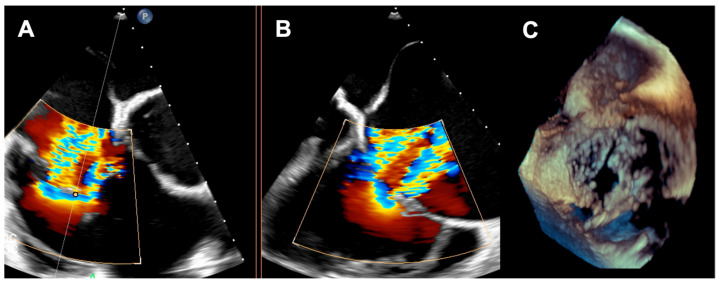
(**A**,**B**) Torrential tricuspid regurgitation in the RV inflow—outflow tract X-plane view; (**C**) large central coaptation deficit in 3D view. RV—right ventricle.

**Figure 4:**
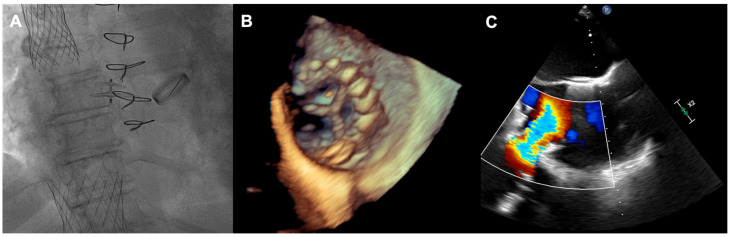
(**A**) Final angiographic result of TricValve implantation; (**B**) 3D view of IVC valve protruding to RA; (**C**) inflow from IVC through the implanted valve. IVC—inferior vena cava; RA—right atrium.

A 73-year-old woman with a history of two surgical interventions—an aortic valve replacement 25 years before and a thoracic aortic aneurysm repair 12 years before—was admitted to the hospital because of persistent heart failure symptoms in functional class NYHA III, accompanied by peripheral edema and ascites. The transthoracic echocardiography showed preserved function of the mechanical aortic prosthesis and torrential TR with tricuspid anulus enlargement. A further transesophageal examination revealed a large, mainly central coaptation deficit between the tricuspid leaflets, located mainly in the central region of the valve (Figure 3). Furthermore, a patent foramen ovale (PFO) with spontaneous right-to-left shunt was also diagnosed. Due to the valve anatomy, the patient was not deemed eligible for the transcatheter edge-to-edge repair. Because of the symptoms’ persistence, a two-step percutaneous procedure was planned. First, the PFO was closed with the 25 mm Amplazer occluder. Two weeks later, the CAVI procedure with the implantation of TricValve system valves (SVC 29 mm; IVC 45 mm) was carried out without complications. (Figure 4) We believed that the PFO closure was necessary before the CAVI procedure because the implantation of venae cavae valves in a patient with a patent PFO would have caused an exacerbation of the right-to-left shunt and possible patient desaturation. In observation, six and twelve months after the procedure, the patient remained on stable doses of oral diuretics and was considered to be in NYHA functional class II, without worsening heart failure or requiring hospitalization.

### 3.3. Case 3

The third patient was a 67-year-old woman with heart failure symptoms in the NYHA III class, accompanied by ascites and peripheral edema that were caused by persistent massive TR. The patient’s past medical history included surgical aortic valve replacement 20 years before, persistent atrial fibrillation, type 2 diabetes, and obesity. The patient’s TR had a mixed origin; it was partially due to an interaction with the right ventricular pacemaker lead. However, it persisted after a percutaneous lead extraction procedure 14 months before the CAVI procedure and subsequent implantation of a leadless pacemaker. After lead extraction, the patient’s tricuspid valve anatomy was not eligible for the T-TEER procedure. TricValve implantation (SVC 25 mm and IVC 31 mm) was carried out without complications; however, the patient’s echocardiography after the procedure showed a mild paravalvular leak in their IVC valve. Three and six months after the procedure, the patient persisted in NYHA functional class II; however, during the following months, she was hospitalized due to exacerbation of right ventricular failure. The patient died 10 months after the procedure because of a worsening of the right heart and multiorgan failure.

### 3.4. Case 4

The last patient was a 66-year-old woman with torrential TR due to carcinoid heart disease in the context of multiple endocrine neoplasia (MEN) syndrome. The patient underwent peptide receptor radionuclide therapy with adjunctive chemotherapy for MEN disease, with confirmed hepatic metastases. The patient’s echocardiographic examination showed an enlarged right ventricle with preserved contractile function and a torrential TR with a large coaptation deficit of 18–20 mm. TricValve implantation (SVC 25 mm and IVC 35 mm) was carried out uneventfully. The patient’s echocardiography, after the procedure, confirmed the proper positioning and functioning of both valves, without a significant paravalvular leak. During the following months after the procedure, the patient’s condition improved and remained stable in NYHA functional class II. However, after a few months, the patient’s carcinoid disease worsened, and the patient died 7 months after the procedure due to progression of neoplastic disease.

## 4. Discussion

The method of heterotopic valvular prostheses placement in the ostium of the SVC and IVC for the treatment of TR symptoms was first proposed by Lauten and co-workers [4]. The procedure prevents regurgitant blood flow into the venous system, and it reduces abdominal congestion, ascites, and peripheral edema. Additionally, by reducing liver congestion, the CAVI is believed to improve liver function. Hemodynamic studies also indicate an increase in right ventricular stroke volume, and thanks to it, overall cardiac output [5]. Out of several devices proposed for the CAVI procedure, the TricValve is the first system that has been accepted for commercial use in the European market. Both SVC and IVC self-expanding valves are premounted in a 27.5-F delivery system. Caval anchoring is based on stent design, radial force, and the degree of oversizing. The selection of valve size is based on the computed tomography measurements of both the SVC and IVC. Multiple measurements are taken at various anatomical landmarks along the SVC and IVC. The length of the SVC and the distance from the IVC to the origin of the hepatic veins are crucial factors in determining whether the device can be accommodated in the SVC or IVC.

Safety and efficacy of the TricValve implantation were the subject of TRICUS and TRICUS EURO studies [6,7]. Those prospective registries recruited patients with at least severe TR in whom NYHA class III and IV heart failure symptoms persisted despite optimal pharmacological treatment. Additionally, observed patients, due to the high risk of intervention, were found to be ineligible for the surgical correction of valvular heart disease. The primary study endpoint was a significant improvement in life quality, defined as at least a 15-point increase in the Kansas City Cardiomyopathy Questionnaire (KCCQ) result, a reduction in symptoms to the NYHA classes less than II, or at least a 40 m increase in distance in the 6 min walking test. The registries included a total of 44 patients (82% women; mean age 76.2+/−7.5 years) with severe heart failure symptoms (86.4% NYHA III, 13.6% NYHA IV). The primary efficacy endpoint was reached in 42 (95.5%) patients. It included at least a 15-point KCCQ increase in 56.4% and a reduction in class NYHA II symptoms or less in 62.2% of study participants. Although the difference in the mean 6 min walking test distance failed to reach the statistical significance level (229+/−91 m at baseline vs. 270+/−111 m in one-year follow-up) in 40% of patients, a substantial improvement, defined as a 40 m increase, was noted. Among the secondary endpoints, a significant tricuspid annulus diameter reduction, as well as a decrease in mean NT-proBNP concentration, were observed in the studied population. During the twelve-month observation, no myocardial infarctions were noted; however, four (9%) cases of stroke were observed, and nine patients (20.4%) suffered from major (mainly gastrointestinal) bleeding episodes.

Recently, the TRIC-BICAVAL registry brought additional data regarding the clinical effectiveness of the TricValve system in an unselected real-life population [8]. This international registry included 204 patients who were recruited in 27 European and Brazilian centers. In the studied group (mean age 77.8+/−7.5 years), 87.3% were diagnosed with massive or torrential TR, and 60.8% had been hospitalized for right ventricular heart failure exacerbation during the year preceding the TricValve implantation. The population was characterized by the presence of multiple risk factors, and predicted mortality during surgical intervention based on the TriScore model, which was calculated at a level of 23.2%. Moreover, half of the study participants had been operated on in the past for valvular heart disease, and a substantial percentage of patients (19.1%) had a history of previous transcatheter valvular intervention. After the implantation of the TricValve system, which was successful in 96.1% of cases, a significant increase of patients in functional NYHA classes I and II was observed (19.8% at baseline vs. 81.5% at 12 months; *p* < 0.001), which was followed by a reported decrease in the heart failure hospitalization rate when compared to the year preceding the intervention (60.8% vs. 25.8%; *p* < 0.001). In this high-risk population, one-year all-cause mortality reached a level of 25%.

Our initial series—patients who were treated with the TricValve system—described above, represents a so-called no-option population of patients. In this heterogenous group with different mechanisms of TR, all of the patients, due to anatomical restraints, were ineligible for the T-TEER procedure, the method most commonly used in transcatheter TR treatment. Additionally, all of the patients were characterized by the risk profile that is prohibitive for surgical intervention—three out of four patients had a history of previous cardiac surgery (two of them twice), and the predicted surgical mortality, based on the TriScore model, was between 14% and 48%. Three procedures were uneventful; one implantation was complicated with the access site bleeding, requiring further surgical intervention. Three out of four patients did not survive the first year after the procedure; two of them died because of heart failure progression, and one died due to underlying neoplastic disease. However, it has to be emphasized that, in all described cases, the CAVI was considered as a palliative method aimed at reducing disabling symptoms that persisted despite pharmacotherapy interventions.

During recent years, we have observed rapid progress in the field of percutaneous TR treatment. Recently, a new method of transcatheter TR therapy was approved. Orthotopic valve placement in the native tricuspid ring with the transcatheter Evoque (Edwards Lifesciences) system was shown to be effective in symptom reduction and quality of life improvement [9]. However, a substantial percentage of referred patients are ineligible for the procedure, mainly due to tricuspid annulus enlargement. In such patients with symptoms persisting despite optimal medical therapy, heterotopic caval valve implantation remains a viable and interesting treatment option. The TricValve procedure might also be helpful in the treatment of patients who are ineligible or not benefiting from other tricuspid interventions. Furthermore, because heterotopic valve implantation does not interfere with the native tricuspid apparatus, it may also be useful in patients with TR that is caused by cardiac implantable devices.

On the other hand, it needs to be emphasized that existing evidence regarding TricValve implantation is based on open-label registries, including a limited population of patients, while data from randomized studies comparing the method to, for example, optimal medical therapy, is still lacking. Furthermore, the CAVI concept itself has several limitations; most importantly, it does not offer a physiological correction of TR. The procedure prevents the backflow of blood to the venous system, leading to right atrium ventricularization. Since the remote effects of such a phenomenon remain unknown, further studies are needed to elucidate the problem in long-term follow-ups. Given its limitations, the device will likely be recommended for a selected group of patients. The current target population for transcatheter therapies for severe TR typically consists of elderly, frail individuals with significant comorbidities and reduced quality of life. For these patients, the primary objectives of care are to enhance their quality of life and reduce hospitalization rates.

## 5. Conclusions

In highly symptomatic TR patients who are ineligible for the T-TEER procedure, and who have a prohibitive risk of cardiac surgery, TricValve implantation led to a reduction in symptoms in a 6-month perspective. Patients’ long-term survival was limited mainly by heart failure progression and severe concomitant disorders. Further studies are needed to fully elucidate the role of caval valve implantation in the treatment of TR patients.

## Figures and Tables

**Table 1:** Patient characteristics and results summary.

	Overall	Case 1	Case 2	Case 3	Case 4
Age	70.5 (67.5–74.0)	77	73	68	66
Sex	100% F	Female	Female	Female	Female
Symptoms (NYHA)	3.0 (3.0–3.0)	III	III	III	III
RV failure symptoms		-Ascites -Peripheral edema	-Ascites -Peripheral edema	-Ascites -Peripheral edema	-Ascites, -Peripheral edema
Euroscore II	9.7% (8.6–12.1%)	17.1%	8.9%	10.47%	7.8%
Triscore	35% (20–48%)	8/12 (48%)	8/12 (48%)	6/12 (22%)	5/12 (14%)
Previous cardiac surgery		CABG; TVR	AVR; thoracic aortic aneurysm	AVR	
Echocardiography					
- LVEF	58.0 (54.5–59.2)	47%	57%	60%	59%
- LVDD	4.9 (4.9–5.0)	4.9 cm	5.1 cm	4.9 cm	4.8 cm
- LA	4.7 (4.4–4.8)	4.8 cm	4.6 cm	4.0 cm	4.8 cm
- RAA	27.5 (25.8–28.8)	30 cm^2^		24 cm^2^	27.5 cm^2^
- RV	4.2 (4.0–4.2)	4 cm	3.7	4.2 cm	4.2 cm
- AcT	99.0 (97.0–114.5)	99 ms	130 ms		95 ms
- TR grade (1–5)	4.5 (4.0–5.0)	4/5	5/5	4/5	5/5
- TAPSE	16.5 (15.0–17.8)	12 mm	16 mm	17 mm	20 mm
- SPAP	41.0 (34.5–43.0)	41 mmHg	45 mmHg		28 mmHg
- IVC	36.0 (30.8–43.8)	41 mm	52 mm	30 mm	31 mm
Laboratory findings					
- NT proBNP	1156.2 (735.5–1673.8)	1493 pg/mL	484 pg/mL	819.4 pg/mL	2216 pg/mL
- eGFR	42.1 (35.0–56.4)	36 mL/min/1.73	77 mL/min/1.73	35.9 mL/min/1.73	55.5 mL/min/1.73
- Bilirubin (total)	1.5 (1.0–17.8)	1.19 mg/dL	1.07 mg/dL	0.6 mg/dL	
Diuretics (daily dose)					
- furosemide	160.0 (100.0–220.0)	280 mg	160 mg	40 mg	-
- torasemide	50.0 (35.0–75.0)	20 mg	-	100 mg	50 mg
- MRA	50.0 (43.8–50.0)	50 mg	50 mg	50 mg	25 mg
- HCTZ	12.0 (8.5–18.5)	25 mg	12.5 mg	-	-
CAVI procedure					
- SVC valve diameter	25.0 (25.0–26.0)	25 mm	29 mm	25 mm	25 mm
- IVC valve diameter	38.0 (34.0–42.0)	41 mm	45 mm	31 mm	35 mm
- complications		-Access site bleeding -IVC paravalvular leak	None	-Mild paravalvular leak in the IVC valve	None

AVR—aortic valve replacement; CABG—coronary artery bypass graft; HCTZ—hydrochlorothiazide; IVC—inferior vena cava; LA—left atrium; LVDD—left ventricle diastolic diameter; LVEF—left ventricle ejection fraction; MRA—mineralocorticoid antagonist; NYHA—New York Heart Association; RV—right ventricle; SVC—superior vena cava; TAPSE—tricuspid anulus plane systolic excursion; TR—tricuspid regurgitation; TVR—tricuspid valve repair.

## Data Availability

The original contributions presented in this study are included in the article. Further inquiries can be directed to the corresponding author(s).
